# Mutual Stabilization of Rhythmic Vocalization and Whole-Body Movement

**DOI:** 10.1371/journal.pone.0115495

**Published:** 2014-12-12

**Authors:** Kohei Miyata, Kazutoshi Kudo

**Affiliations:** Department of Life Sciences, Graduate School of Arts and Sciences, The University of Tokyo, Tokyo, Japan; University of California, Merced, United States of America

## Abstract

The current study investigated the rhythmic coordination between vocalization and whole-body movement. Previous studies have reported that spatiotemporal stability in rhythmic movement increases when coordinated with a rhythmic auditory stimulus or other effector in a stable coordination pattern. Therefore, the present study conducted two experiments to investigate (1) whether there is a stable coordination pattern between vocalization and whole-body movement and (2) whether a stable coordination pattern reduces variability in whole-body movement and vocalization. In Experiment 1, two coordination patterns between vocalizations and whole-body movement (hip, knee, and ankle joint flexion-on-the-voice vs. joint extension-on-the-voice) in a standing posture were explored at movement frequencies of 80, 130, and 180 beats per minute. At higher movement frequencies, the phase angle in the extension-on-the-voice condition deviated from the intended phase angle. However, the angle of the flexion-on-the-voice was maintained even when movement frequency increased. These results suggest that there was a stable coordination pattern in the flexion-on-the-voice condition. In Experiment 2, variability in whole-body movement and voice-onset intervals was compared between two conditions: one related to tasks performed in the flexion-on-the-voice coordination (coordination condition) that was a stable coordination pattern, and the other related to tasks performed independently (control condition). The results showed that variability in whole-body movement and voice-onset intervals was smaller in the coordination condition than in the control condition. Overall, the present study revealed mutual stabilization between rhythmic vocalization and whole-body movement via coordination within a stable pattern, suggesting that coupled action systems can act as a single functional unit or coordinative structure.

## Introduction

Rhythmic auditory stimulation (RAS) is expected to be a means for enhancing performance in practical settings, particularly gait rehabilitation and sports. With respect to gait rehabilitation, extensive clinical studies have shown that RAS improves several aspects of gait timing among patients with Parkinson's disease, such as gait tempo, stride length, and the magnitude of stride-time variability [1]–[6]. These facilitating effects of RAS on gait performance have also been reported in patients with hemiparetic stroke [7] and traumatic brain injury [4]. In terms of sports, previous research demonstrated that RAS enhances performance in sports that are cyclic in nature such as running and cycling [8], [9]. In regard to running, Bood et al. [8] reported that time to exhaustion was longer with metronome beats than without. RAS also reduced energy costs during cycling [9]. One of the reasons for enhancing performance due to RAS relates to movement stabilization. Rhythmic acoustic stimuli generated by external devices are more stable than human movement so that movement stabilization occurs when accompanied with external acoustic stimuli, such as metronome beats.

Meanwhile, not only external devices but also vocalizations can generate rhythmic acoustic stimuli. One question is whether vocalizations that generate rhythmic acoustic stimuli could be a possible alternative to metronome beats for enhancing performance through movement stabilization. Concerning interactions between concurrent vocalizations and movements, several studies have suggested an interfering effect of vocalizations on motor performance [10]–[15]. For example, Hiscock and Chipuer [11] asked participants to rhythmically tap a telegraph key while reciting verbal sentences that had an irregular rhythm; they found that variability in the tapping interval increased due to the concurrent vocalization. This suggested there was interference between speech and manual tapping. Inhoff and Bisiacchi [12] also reported that vocalizations with relatively complex articulation increase tapping variability. These studies suggest that dual-task interference occurs between vocalizations and manual task performance. However, more recent studies on coordination dynamics indicate that an interaction between vocalizations and other active effectors can be understood as being coupled and coordinated systems [16]–[18]. In this dynamical view, while coupled systems can behave with large fluctuations in terms of relative timing as reported by studies on dual-task interference [11], [13], they can also act as a single functional unit or coordinative structure through functional coupling or mutual constraint between the systems [19]. Previous studies have suggested that behaving as a coordinative structure can decrease temporal variability [20], [21]. For example, Helmuth and Ivry [20] reported reduced variance during bimanual as compared to unimanual finger tapping, referred to as the bimanual advantage. They also demonstrated that this bimanual advantage was obtained during tapping movements with non-homologous effectors such as the finger and forearm. Therefore, it can be hypothesized that concurrent vocalizations that are well coupled with rhythmic movement can decrease the variability of that rhythmic movement, and vocalization as well.

Human rhythmic coordination, such as auditory-motor, inter-limb, and intra-limb coordination, has often been investigated in the context of the phase transition paradigm. In this paradigm, increasing movement frequency plays a critical role in the organization of human coordination patterns [22]–[24]. At low movement frequencies, there are no significant differences in the stability of two distinct coordination patterns, such as in-phase vs. anti-phase, synchronization vs. syncopation, or flexion-on-the-beat vs. extension-on-the-beat, during human rhythmic movements. However, as movement frequency increases, a spontaneous transition occurs from a less stable coordination pattern to a more stable one. This transition is called *phase transition*. Miura et al. [23], [24] investigated metronome beats and whole-body movement coordination involving flexing and extending one's knees repeatedly in a standing posture with metronome beats. They demonstrated that increased movement frequency induced phase transition from an extension-on-the-beat to a flexion-on-the-beat coordination pattern. This can be understood as there being two *attractors* during both coordination patterns at a low movement frequency. From there, the extension-on-the-beat *attractor* was lost due to incremental movement frequency, and a transition to the flexion-on-the-beat occurred. This also indicates that the flexion-on-the-beat is a stable coordination pattern. This phenomenon have also been observed during inter-limb [25]–[27] and intra-limb coordination [28], [29]. These findings suggest that rhythmic movement coordination is governed by general dynamical principles.

In the example of the bimanual advantage [20], the phase relation of bimanual tapping is in-phase (i.e., a stable coordination pattern during inter-limb coordination). When investigating movement stabilization due to rhythmic vocalization, it is necessary to investigate the property of coordination between vocalizations and rhythmic movement to reveal a stable coordination pattern. Previous studies have reported a stable coordination pattern during auditory-motor [22]–[24], inter-limb [25]–[27], and intra-limb coordination [28], [29]. Therefore, it can be hypothesized that there is also a stable coordination pattern between vocalizations and movement. Indeed, Treffner and Peter [18] demonstrated that transition from anti-phase coordination (syncopated or alternated speech-tapping) to in-phase coordination (synchronized speech-taping) occurred during a speech-hand coordination task when a metronome frequency as a pacing signal increased. Since they used metronome beats to manipulate movement frequency, a stable coordination pattern was induced by the external pacing signal.

The central goal for the current paper was to investigate whether variability in rhythmic movement is reduced through coordination with vocalizations within a stable coordination pattern. We conducted two experiments. The aim of the first experiment was to examine whether there is a stable coordination pattern between vocalizations and movement without metronome beats. The current study used similar methods from a previous studies by Miura et al. [23], [24], including a whole-body movement task, pace manipulation, and analyses. We used two kinds of coordination patterns between whole-body movement and vocalizations in a standing posture: flexion-on-the-voice condition (hip, knee, and ankle joint flexion with vocalization) and extension-on-the-voice condition (joint extension with vocalization). We expected that when movement frequency was increased, relatively unstable coordination pattern was replaced by a stable coordination pattern. The aim of the second experiment was to investigate whether variability in whole-body movement and vocalizations decreases through coordination during a stable coordination pattern. We focused on temporal stability and regarded decreases in movement variability as increases in movement stability. The variability of vocalizations and whole-body movement was assessed by the standard deviation (SD) of the interval and compared between two conditions; one condition included the tasks performed in a stable coordination pattern (coordination condition), and the other included tasks being performed independently (control condition). We expected that variability of the two movements would be smaller in the coordination condition than in the control condition.

## Experiment 1

### Methods

#### Participants

Fourteen healthy volunteers (14 males; aged 23–36 years) participated in this experiment. All had no known neurological or movement deficits and were non-dancers recruited from the University of Tokyo community. All participants signed an informed consent document before participating. The Ethics Committee of the Graduate School of Arts and Sciences, the University of Tokyo, approved this study.

#### Experimental task

**Figure 1:**
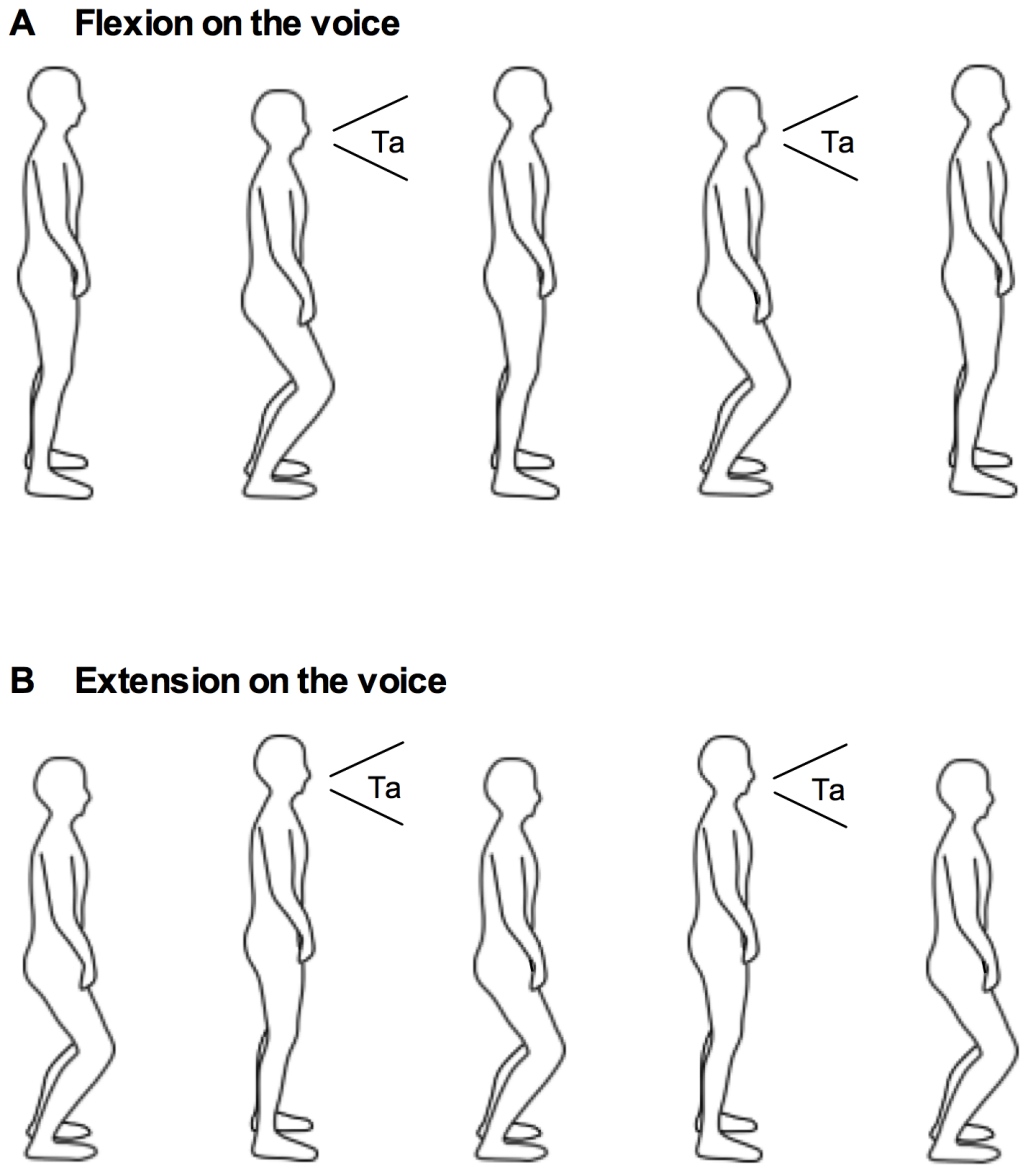
Flexion on the voice condition (A) and extension on the voice condition (B). In the flexion-on-the-voice condition, participants flexed their hip, knee, and ankle joints in line with a vocalization (A). In the extension-on-the-voice condition, participants extended their joints in line with a vocalization (B).

The task included a whole-body movement of flexing and extending one's hip, knee, and ankle joints repeatedly to go up and down one's center of mass while maintaining a standing posture. The task also included the vocalization “ta” at a 1∶1 frequency locking with the whole-body movement. Participants were instructed to stand with their feet roughly at shoulder width and look at a fixation point on a wall at a distance of 1 m. The repetitive flexing and extending movement was the same as in previous studies [23], [24]. Participants were asked to flex and extend their hip, knee, and ankle joints in a comfortable angular range without their feet leaving the ground. The repetitive vocalization was performed at each participant's usual speech volume. Although participants were asked to continue to vocalize as much as they could, they were allowed to temporarily stop the vocalization if they wanted to breathe. Two different coordination patterns were investigated: flexion-on-the-voice and extension-on-the-voice (Fig. 1). In the flexion-on-the-voice condition, participants flexed their hip, knee, and ankle joints in line with a vocalization (Fig. 1-A). In the extension-on-the-voice condition, participants extended their joints in line with a vocalization (Fig. 1-B). The phase transition paradigm needs to manipulate movement frequency continuously within a trial. Since it is difficult for participants to control movement frequency systematically without metronome beats, we aimed at capturing a stable coordination pattern and manipulated movement frequency in separate trials. The movement frequency was set at 80, 130, and 180 beats per minute (bpm), and task duration was approximately 35, 25, and 20 seconds, respectively. There were approximately 40 cycles for each flexion and extension trial. Before each recording, participants listened to metronome beats as a reference for movement frequency. Then participants performed the tasks at the memorized frequency without auditory signals to help with pacing. Trials where the average frequency was within ±20 bpm of the assigned frequency were defined as successful trials. As a result, approximately 4% of all trials were removed and re-recorded. During task performance, participants were instructed not to move any other parts of their body other than the hip, knee, and ankle joints.

#### Design and procedure

Participants went through 18 trials (2 coordination patterns ×3 movement frequencies ×3 repetitions). The first 3 cycles were discarded to remove unstable coordination, and subsequent 30 cycles were analyzed for each participant. We also conducted the analysis with the inclusion of all cycles and checked the consistent results. Trial order was randomized. Before the experimental session, participants practiced the tasks for a few minutes. Participants were instructed to keep a one-to-one correspondence between their whole-body movement and vocalizations and not resist any pattern change; additionally, they were asked to establish the most comfortable pattern. Participants had brief breaks between trials in order to limit fatigue.

#### Data collection and analysis

**Figure 2:**
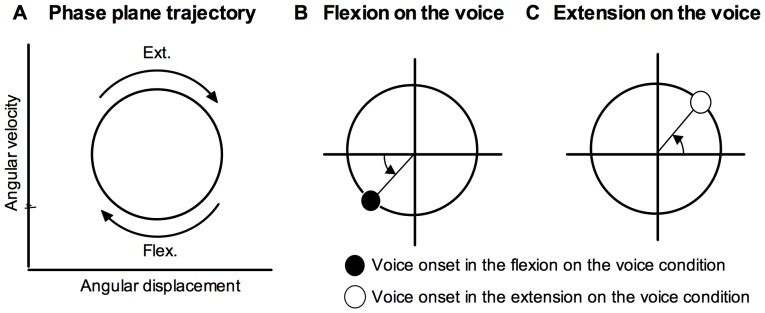
Knee movement trajectory and voice time on the phase plane. Knee movement trajectory on the phase plane (A). The phase angle is expressed as the difference from the peak knee position. In the flexion-on-the-voice condition, difference between voice time and the peak knee-flexion point is calculated (B), and in the extension-on-the-voice condition, difference between voice time and the peak knee-extension point is calculated (C).

Knee angular displacement as a representative of joint motion of the extension-flexion movement was measured using an electro goniometer (Biometrics Ltd, SG150, Newport, United Kingdom) attached to the right knee joint. Vocalization was measured using a headset microphone (SHURE, WH20XLR, Chicago, USA) and amplified (audio-technica, AT-MA2, Tokyo, Japan). All data were digitized at 1 kHz using an analog-digital converter (ADI Instrument, PowerLab 4/25 s, NSW, Australia). Angular displacement data were filtered using a low-pass filter (cutoff frequency = 7 Hz). The cutoff frequency was determined from a residual analysis of the difference between the filtered (at cutoff frequencies in the range 1–20 Hz) and the unfiltered data [30]. Angular velocities were obtained by differential calculus of angular displacement data. Angular displacement and angular velocity were normalized and plotted on the phase plane (Fig. 2-A). The vocal onset times were defined when voice amplitude exceeded a threshold set at approximately 20% of the average peak value and superimposed on the phase plane trajectory. Vocal onsets in the flexion-on-the-voice (Fig. 2-B) and extension-on-the-voice (Fig. 2-C) conditions were expressed in solid and open dots, respectively. The relative phase angle between the knee movement and voice, and its SD, were calculated using circular statistics [31]. In the flexion-on-the-voice condition, the phase angle was calculated from the peak knee flexion point (Fig. 2-B); in the extension-on-the-voice condition, the angle was calculated from the peak knee extension point (Fig. 2-C).

#### Statistical analysis

Two-way analyses of variance (ANOVAs) with two within-subjects factors, coordination pattern (flexion on the voice and extension on the voice) and movement frequency (80, 130, and 180 bpm), were performed on mean voice frequency, mean SD of the voice frequency, and mean SD of the phase angle. Since the phase angle of voice time in the extension-on-the-voice condition did not have a normal distribution at 130 bpm, a Wilcoxon matched-pairs signed rank test was performed at 130 bpm to compare the coordination patterns. Separate ANOVAs were performed on phase angle of voice time at 80 and 180 bpm. If Mauchly's test of sphericity was significant, then the Greenhouse-Geisser corrections were used to identify the significance of the main effects and interactions. The statistical significance level was set at p<.05.

### Results

#### Voice Frequency

**Figure 3:**
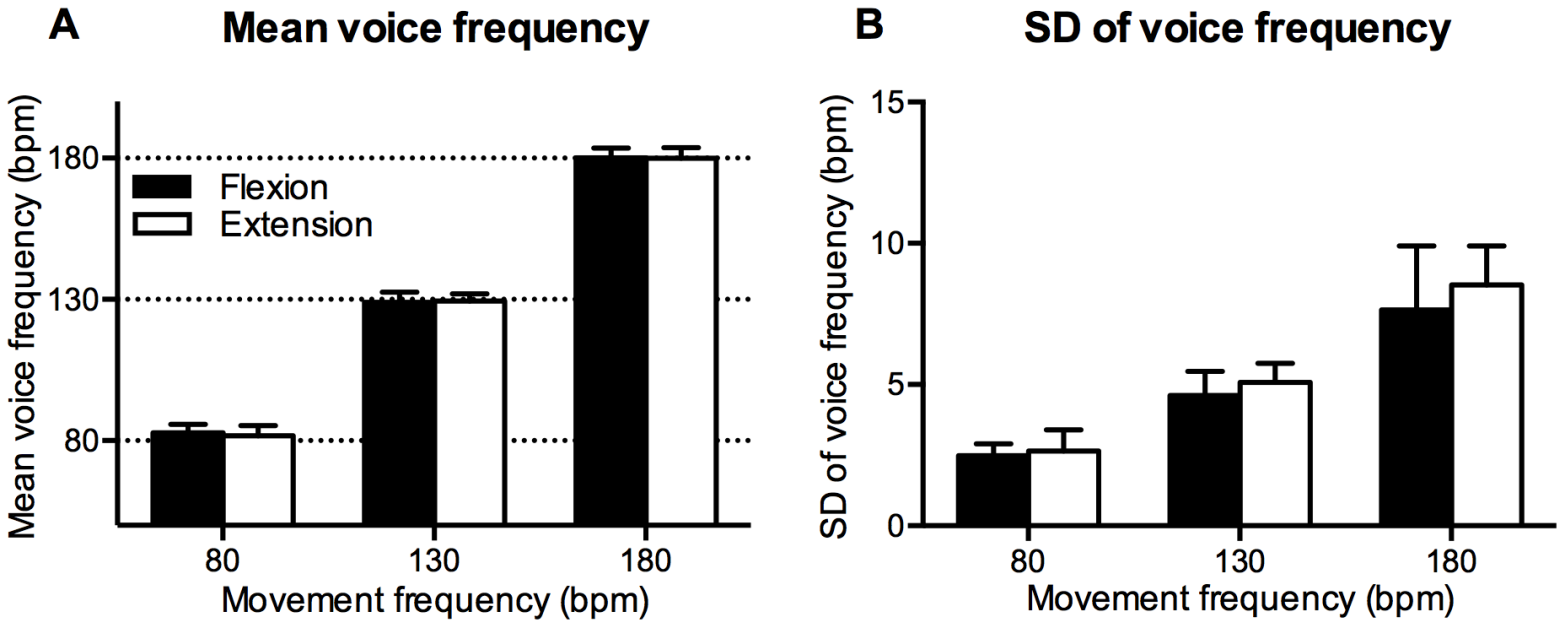
Mean voice frequency (A) and SD of voice frequency (B). Vertical bars represent between-subjects standard deviations.

Voice frequency mean and SD were measured in order to determine whether participants performed tasks at the appropriate frequency. Fig. 3-A shows mean voice frequency. A two-way ANOVA revealed a significant main effect of movement frequency, F (2, 26) = 2862.1, p<.001, but the main effect of coordination pattern was not significant, F (1, 13) = .39, p = .54. The two-way interaction was also not significant, F (2, 26) = .92, p = .41. The mean voice frequency was approximately the same for the specified voice frequency and was not significantly different between the flexion-on-the-voice and extension-on-the-voice conditions.

Fig. 3-B shows the SD of voice frequency. A two-way ANOVA revealed a significant main effect of movement frequency, F (1.11, 14.42) = 113.98, p<.001, and coordination pattern, F (1, 13) = 6.55, p = .02. However, the two-way interaction was not significant, F (1.31, 16.99) = 1.19, p = .31. The SD of voice frequency in the extension-on-the-voice condition was significantly larger than that in the flexion-on-the-voice condition.

#### Phase angle of voice time

**Figure 4:**
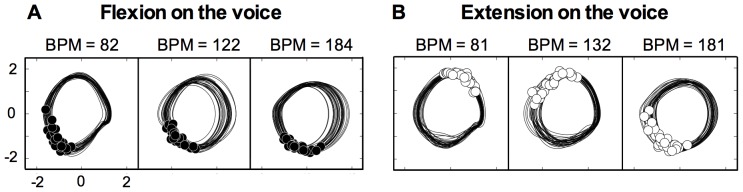
Typical examples of the phase angle of voice time for one participant. The value at the top of each trajectory is the average voice frequency in each trial. In the extension-on-the-voice condition (B), the replacement of joint extension on the voice by joint flexion on the voice occurred at 180 bpm. This replacement did not occur in the flexion-on-the-voice condition.

Fig. 4 shows typical examples of the phase plane trajectories and vocal onsets for one participant. In the flexion-on-the-voice condition, participants performed this task successfully for all movement frequencies (Fig. 4-A). In contrast, in the extension-on-the-voice condition, replacement of knee extension on the voice by knee flexion on the voice occurred at a movement frequency of 180 bpm (Fig. 4-B).

**Figure 5:**
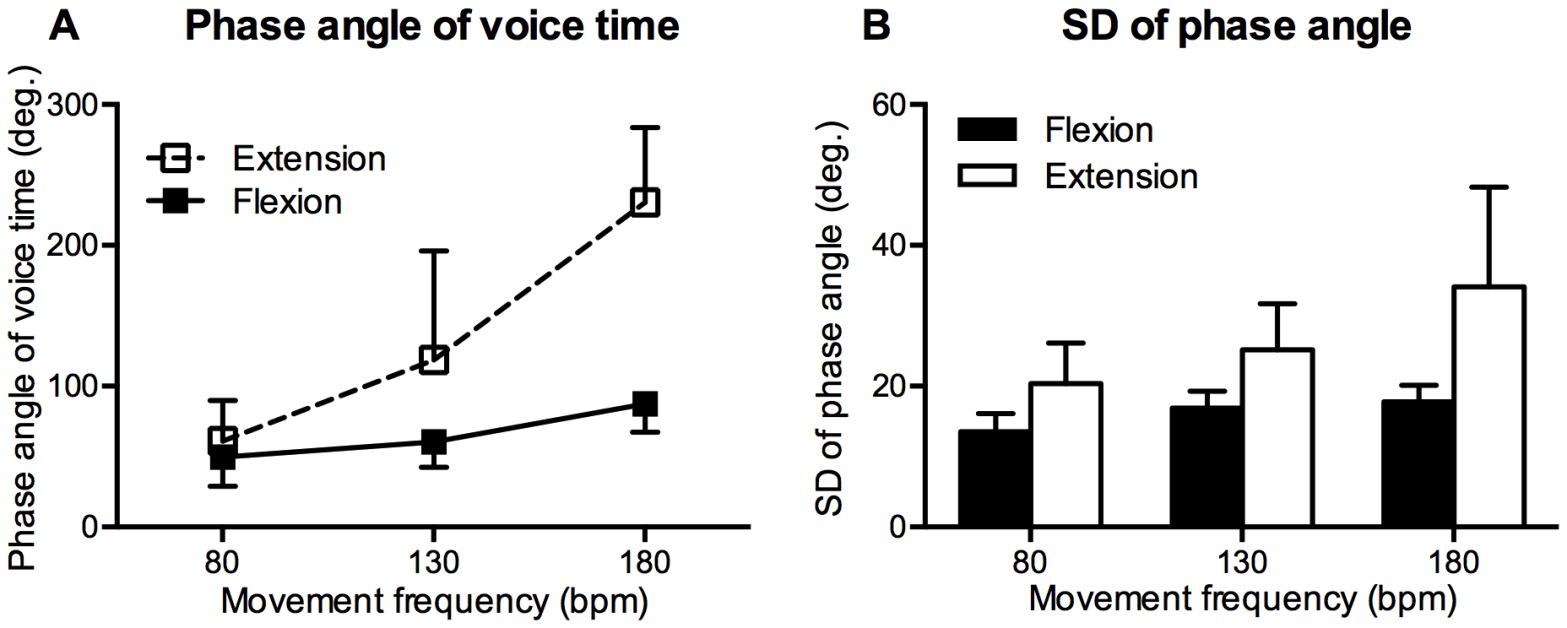
Phase angle of voice time (A) and SD of phase angle (B). Vertical bars represent between-subjects standard deviations.

The phase angle of voice time was calculated to quantify the relationship between voice and knee movement. Fig. 5-A shows the mean phase angle of the voice time as a function of movement frequency. The phase angle of the voice time in the extension-on-the-voice condition was significantly larger than that in the flexion-on-the-voice condition for all movement frequencies of 80, 130, and 180 bpm, F (1,13) = 5.7, p = .03; p = .01; F (1,13) = 153.65, p<.001, respectively.

Fig. 5-B shows the SD of the phase angle as a function of movement frequency. A two-way ANOVA revealed a significant main effect of movement frequency, F (1.2, 15.58) = 13.28, p<.01, and coordination pattern, F (1, 13) = 54.8, p<.001. However, the two-way interaction was not significant, F (1.12, 14.59) = 4.05, p = .06. The SD of the phase angle in the flexion-on-the-voice condition was significantly smaller than in the extension-on-the-voice condition.

### Discussion

Experiment 1 investigated whether there is a stable coordination pattern between vocalizations and whole-body movement. Participants conducted two types of coordination patterns between a vocalization and whole-body movement: hip, knee, and ankle joint flexion with a vocalization and joint extension with a vocalization at different movement frequencies. As movement frequency increased, the phase angle of voice time in the extension-on-the-voice condition shifted away from the required phase angle. Contrary to the extension-on-the-voice condition, the phase angle in the flexion-on-the-voice condition did not show this shift. Furthermore, the SD of the phase angle in the flexion-on-the-voice condition was smaller than that in the extension-on-the-voice condition. These results suggest that the flexion-on-the-voice coordination pattern was more stable than was the extension-on-the-voice coordination pattern.

Experiment 1 did not use metronome beats to manipulate movement frequency while participants performed the tasks. In order to verify that these results were not due to differences in movement frequency, we calculated the mean voice frequency and the SD of voice frequency. Although the SD of voice frequency in the extension-on-the-voice condition was larger than in the flexion-on-the-voice condition, the mean voice frequency was not significantly different between the conditions, suggesting that participants performed the tasks successfully and vocalized with similar frequency in both conditions.

A stable coordination pattern in vocalization and whole-body movement coordination was expected from previous studies on auditory-motor [22]–[24], inter-limb [25]–[27], and intra-limb coordination [28], [29]. Actually, a stable coordination pattern has been demonstrated between vocalizations and finger tapping, although metronome beats were used as a pacing signal [18]. Thus, there remained the possibility that the stable coordination pattern was induced by the external pacing signal. Our results showed a stable coordination pattern between vocalization and whole-body movement without metronome beats. This result suggests that vocalization and movement coordination obeys general dynamical principles and provides additional evidence that human movement coordination is governed by general dynamical principles.

In previous studies assessing various forms of human coordination [22]–[29], movement frequency plays a critical role in the transition of coordination patterns. In the present experiment, a similar phenomenon was observed. The increased movement frequency induced the deviation of the phase angle in the extension-on-the-voice condition from the intended phase angle but not in the flexion-on-the-voice condition. In typical examples of phase plane trajectories and vocal onsets for one participant in the extension-on-the-voice condition (Fig. 4-B), the voice onsets settled into the flexion-on-the-voice coordination pattern at 180 bpm. The group data for the phase angle of voice time also showed that the phase angle at 180 bpm in the extension-on-the-voice condition was the flexion-on-the-voice coordination pattern because the angle at 180 bpm was about 180 degrees greater than at 80 bpm (Fig. 5-A). Although we did not use the phase transition paradigm, a phase transition from the extension-on-the-voice to the flexion-on-the-voice coordination pattern was estimated from our results and examples of phase transitions in previous studies [22]–[29].

## Experiment 2

### Methods

#### Participants

The same 14 participants from Experiment 1 participated in Experiment 2.

#### Experimental task

Participants performed two tasks: one was flexing and extending their hip, knee, and ankle joints repeatedly without vocalization; the other task was to repeat the vocalization “ta” without any body movement. The movement frequencies were set at 80, 130, and 180 bpm, and task durations were approximately 35, 25, and 20 seconds, respectively. The number of flexing and extending cycles or vocalizations during each trial was approximately 40. Before each recording, participants listened to metronome beats as a reference frequency for the task. Then, participants performed the tasks at the memorized frequency without auditory pacing signals. Thus, trials where the average frequency was within ±20 bpm of the assigned frequency were defined as successful trials. As a result, approximately 4% of all trials were removed and re-recorded.

#### Design and procedure

Participants performed 18 trials (2 tasks×3 movement frequencies ×3 repetitions) on a different day from Experiment 1 to preclude any memory effects of the vocalization, which could affect performance. Furthermore, the whole-body movement task was also conducted before the vocalization task. The time interval between the 2 experiments was at least 60 days. Trial order was randomized for each task. The first 3 cycles of each trial were discarded to remove unstable coordination. The subsequent 30 flexing and extending cycles or vocalizations were analyzed for each trial. We also conducted the analysis with the inclusion of all cycles and checked the consistent results.

#### Data collection and analysis

The apparatus and data recording were the same as in Experiment 1. For Experiment 2, we focused on temporal stability. In order to investigate whether the variability of whole-body movement and vocalizations was reduced when two movements are coordinated during a stable coordination pattern (flexion-on-the-voice), the variability of whole-body movement and vocalizations was compared between two conditions (i.e., control vs. coordination condition). Participants were asked to repeat hip, knee, and ankle joint flexion and extension in a standing position without vocalization or repeat vocalizations without any body movement in the control condition. In the coordination condition, participants were instructed to coordinate hip, knee, and ankle joint flexion with a vocalization. Variability of whole-body movement was evaluated by the SD of the peak knee flexion interval (*I_knee_*) measured by calculating the time interval between knee flexion peaks. Variability in vocalizations was also compared between the two conditions, which was evaluated by the SD of the vocal onset interval (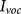) measured by calculating the time interval between vocal onsets. Knee movement and vocalization data in the flexion-on-the-voice condition recorded in Experiment 1 were used for the coordination condition in Experiment 2. The data of knee movement and vocalization recorded in Experiment 2 were used as the control condition. The order of conditions was not randomized so that order and learning effects could affect results.

#### Statistical analysis

Two 2-way ANOVAs with one between-subjects factor (condition: control and coordination) and one within-subjects factor (movement frequency: 80, 130, and 180 bpm) were performed on the SD of *I_knee_* and 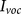. If the two-way interaction was significant, we performed separate post-hoc ANOVAs for movement frequency. If Mauchly's test of sphericity was significant, then Greenhouse-Geisser corrections were used to identify the significance of the main effect and interaction.

### Results

#### SD of *I_knee_* and *I_voc_*

**Figure 6:**
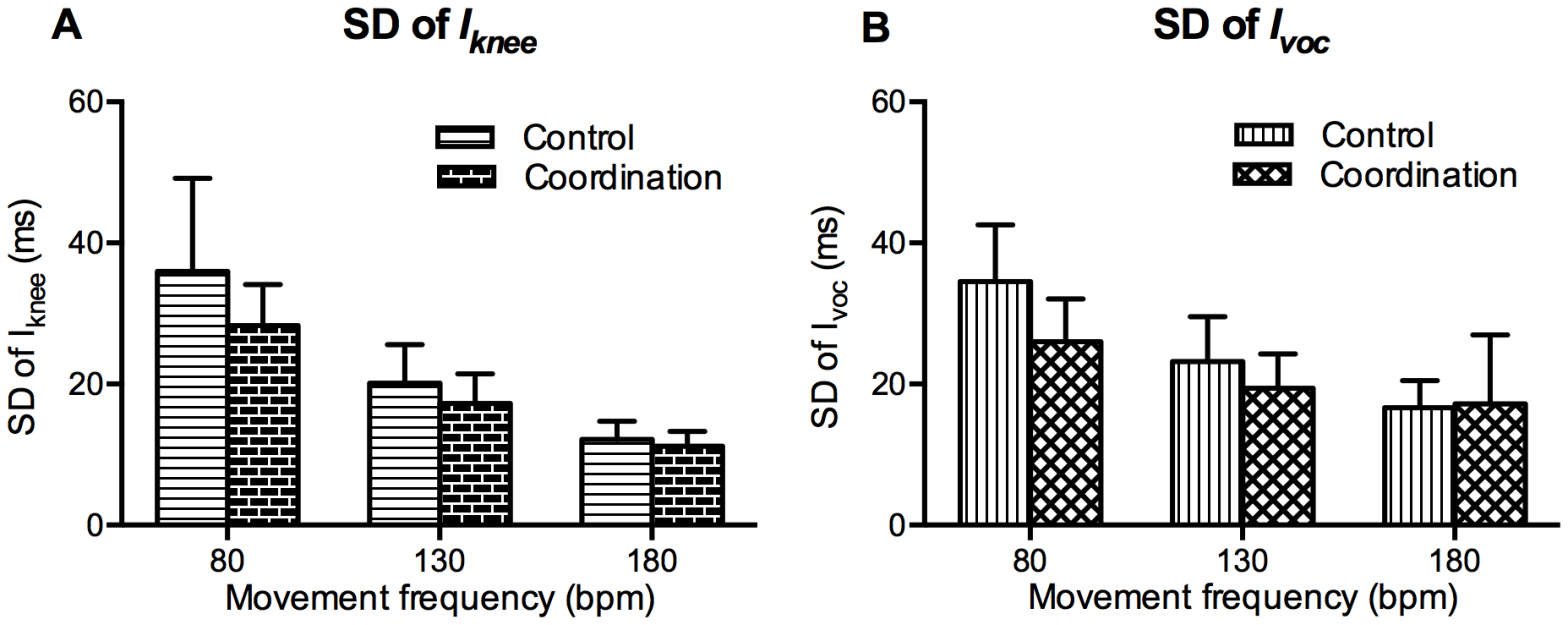
SD of 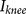 (A) and 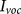 (B). Vertical bars represent between-subjects standard deviations.

In order to investigate the effect of coordination with vocalizations on the variability of whole-body movement, the SD of *I_knee_* was calculated and compared among conditions (Fig. 6-A). We also computed the SD of *I_voc_* in order to explore the effect of coordination with whole-body movement on the variability of vocalizations (Fig. 6-B).

Fig. 6-A shows the SD of *I_knee_*. A two-way ANOVA revealed significant main effects of movement frequency, F (1.42, 36.81) = 83.64, p<.001, and condition, F (1, 26) = 5.4, p = .03. However, the two-way interaction was not significant, F (1.42, 36.81) = 2.59, p = .1. The SD of *I_knee_* in the coordination condition was significantly smaller than that in the control condition.

Fig. 6-B shows the SD of *I_voc_*. A two-way ANOVA revealed a significant two-way interaction, F (2, 52) = 4.25, p = .02, and separate post-hoc ANOVAs were performed to compare the control and coordination conditions at each movement frequency. The SD of *I_voc_* in the coordination condition was significantly smaller than that in the control condition at 80 bpm, F (1, 26) = 9.92, p<.01. Although it was not significant, the SD of *I_voc_* in the coordination condition was also smaller than that in the control condition at 130 bpm, F (1, 26) = 3.11, p = .09.

### Discussion

Experiment 2 investigated whether the variability of whole-body movement and vocalizations is reduced when two movements are coordinated in a stable coordination pattern (flexion-on-the-voice). In order to evaluate the variability of whole-body movement, the SD of *I_knee_* was calculated and compared between two conditions: control condition (hip, knee, and ankle joint flexion without vocalization) and coordination condition (joint flexion on the voice). The SD of *I_knee_* in the coordination condition was smaller than in the control condition. We also compared the SD of *I_voc_* in the two conditions. The SD of *I_voc_* in the coordination condition was also smaller than in the control condition at 80 and 130 bpm. At 180 bpm, the SD of *I_voc_* did not differ between the two conditions. This might be because the vocalization interval was short at 180 bpm in which vocalizations could be perturbed occasionally for respiration or gas exchange. We discuss a possible explanation for the increased stability of rhythmic vocalizations and whole-body movement in General Discussion.

Trials in the coordination and control conditions were not randomized to exclude any memory of vocalizations, which could affect performance. However, in studies where conditions are not randomized, order and learning effects could affect obtained results. Our results showed that the SD of 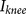 and 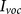 in the control condition was larger than in the coordination condition. These results demonstrated that learning effects were likely negligible since performance conducted 60 days later actually diminished rather than improved.

## General Discussion

The goals of the present study were, first, to examine whether there is a stable coordination pattern between vocalizations and whole-body movement and, second, to explore whether the variability of whole-body movement and vocalizations is reduced when two movements are coordinated in a stable coordination pattern. We revealed that the flexion-on-the-voice coordination pattern was stable, and the SD of 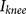 and 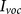 in the coordination condition was smaller than that in the control condition. This suggests that vocalization and whole-body movement strengthened each other's companion stability.

Several studies have reported that vocalization has an interfering effect on motor performance [10]–[15], suggesting that dual-task interference occurs between concurrent vocalizations and manual activity. Previous dual-task studies have reported interference, which refers to decrements in performance for one or both tasks when two activities are carried out concurrently [32], [33]. Pellecchia and Turvey [32] reported that errors on a bimanual coordination task increased when coupled with arithmetic tasks. In the present study, performing vocalizations and whole-body movement concurrently might also be a dual-task. However, results showed that each rhythmic movement was not detrimentally affected, even though each movement was performed as part of a dual-task. Pellecchia [33] reported that dual-task training, but not single-task training, reduced dual-task interference. This finding suggests that strengthening the linkage between the component tasks is important. In line with this suggestion, studies on coordination dynamics indicate that an interaction between vocalizations and other active effectors can be well understood as coupled systems [16]–[18]. Movement stabilization through acting as a coordinative structure has been reported in previous studies [20], [21]. The results also showed the variability of vocalizations and whole-body movement coordination was reduced when being coordinated in a stable manner.

Vocalization and whole-body movement coordination differs from inter-limb and intra-limb coordination in that vocalization has two aspects of movement and auditory feedback. Drewing and Aschersleben [21] have reported that the bimanual advantage is due to an increase in sensory information such as proprioceptive sensibility. That is, timing is better as more sensory information becomes available. Therefore it can be expected that vocalizations and movement coordination is more stable than inter-limb and intra-limb coordination. On the other hand, Helmuth and Ivry [20] argued that there are different timing signals associated with each limb, and the bimanual advantage is due to output integration of effector-specific timers. They explained that the unimanual and bimanual conditions can be considered as two pendulums of different mass. The smaller pendulum can represent the unimanual condition. The larger pendulum can represent the bimanual condition, where two small pendulums have been strictly coupled (and thus have larger mass). The larger pendulum is less subject to a perturbation than is the smaller one, indicating that the bimanual condition has reduced variability. In case of that, there would be no advantage of vocalizations and movement coordination. Future research we should address these two possibilities, increased sensory information and the integration of effector-specific timer output.

There might be stabilization mechanisms specific to the interaction between vocalizations and motor performance based on neurophysiological evidence. For instance, increases in excitability of corticospinal pathways toward dominant hand muscles due to vocalizations has been reported in previous studies [34], [35], suggesting that vocalizations facilitate hand movement. On the other hand, Broca's area, the cortical area for speech production and articulation, is also activated by hand movement [36]. These neurophysiological results indicate mutual facilitation between vocalizations and hand movement. Although our motor task was more global than simply hand movements, mutual facilitation between vocalizations and whole-body movement might appear as mutual stabilization.

A stable coordination pattern between vocalizations and whole-body movement indicates coordination between voices as acoustic stimuli and/or vocalizations as movement and whole-body movement. A stable coordination pattern between external acoustic stimuli and rhythmic movement has been reported in previous studies assessing auditory-motor coordination [22]–[24]. Thus, a stable coordination pattern between voices as acoustic stimuli and whole-body movement should occur. The possibility of a stable coordination pattern between vocalizations as movement and whole-body movement is also suggested by previous studies assessing inter-limb [25]–[27] and intra-limb coordination [28], [29]. Furthermore, mutual stabilization between vocalizations and whole-body movement, also indicate voices as acoustic stimuli and/or vocalizations as movement stabilize whole-body movement. In the future, we plan to investigate whether there is a stable coordination pattern and mutual stabilization between vocalizations as movement and whole-body movement. This will be done by obstructing or manipulating auditory feedback to reveal each effect of vocalizations as acoustic stimuli and movement.

Previous studies have reported that various types of motor performance are enhanced by RAS [1]–[9]. The present results are relevant for practical exercise support without the aid of an external device. Our results suggest the possibility that motor performance could be enhanced through vocalizations. Exercise support using vocalizations has been conducted, and performance enhancement on certain motor tasks has been reported in previous studies [37], [38]. However, this past research was conducted with older adults and patients with hemiparetic stroke while examining discrete and partial movement. Our results showed an effect of vocalizations among healthy young men while performing continuous and whole-body movement. In the future, we will investigate the usability of vocalizations for motor learning and clinical settings such as gait rehabilitation.

## Conclusions

Performing two tasks concurrently is typically more difficult than performing a single task because mechanical degrees of freedom that cause control complexity are increased. Our results, however, revealed that performance on two tasks is better in terms of temporal variability than that in individual tasks, suggesting that coupled action systems acting as a single functional unit or coordinative structure could enhance performance. That is, certain motor tasks are stabilized via coordination with other actions, as with vocalizations.
